# The effect of educational intervention on use of psychotropics in defined daily doses and related costs – a randomized controlled trial

**DOI:** 10.1080/02813432.2022.2074055

**Published:** 2022-05-12

**Authors:** Aalto Ulla L, Rantsi Mervi, Juola Anna-Liisa, Kautiainen Hannu, Pitkälä Kaisu H

**Affiliations:** a Department of Social Services and Health Care, City of Helsinki, Helsinki, Finland; b Department of General Practice, University of Helsinki, Helsinki, Finland; c Department of Health and Social Management, University of Eastern Finland, Kuopio, Finland; d Helsinki University Hospital, Unit of Primary Health Care, Helsinki, Finland

**Keywords:** Psychotropics, staff training, defined daily dose, drug cost, long-term care, older adults

## Abstract

**Objective:**

To investigate the effect of an educational intervention of nursing staff on change in psychotropic use and related costs among older long-term care residents.

**Design:**

A secondary analysis of a randomized controlled intervention study with 12 months of follow-up.

**Setting:**

Assisted living facilities in Helsinki, Finland.

**Subjects:**

Older (≥65 years) residents (*N* = 227) living in assisted living facility wards (*N* = 20) in Helsinki in 2011.

**Intervention:**

The wards were randomized into two groups. In one group, the nursing staff received training on appropriate medication therapy and guidance to recognize potentially harmful medications and adverse effects (intervention group); in the other group, the nursing staff did not receive any additional training (control group).

**Main outcome measures:**

Change of psychotropic use counted as relative proportions of WHO ATC-defined daily doses (rDDDs) among older long-term care residents. In addition, the change in drug costs was considered. Comparable assessments were performed at 0, 6, and 12 months.

**Results:**

A significant decrease in both rDDDs and the cost of psychotropics was observed in the intervention group at 6 months follow-up. However, at 12 months, the difference between the intervention and control group had diminished.

**Conclusions:**

Educational training can be effective in reducing the doses and costs of psychotropics. Further studies are warranted to investigate whether long-term effects can also be achieved by various educational interventions.

**Registration number:**

ACTRN 12611001078943

**KEY POINTS**
- We explored the effect of staff training on psychotropic use and associated costs among older long-term care residents.
- Educational training of nursing staff was beneficial as regards the actual drug doses of psychotropics, and cost savings in psychotropic medication were achieved.
- Educational training was efficient in the short-term, but further research is warranted to achieve long-term effects.

## 1. Introduction

Psychotropics are generally considered potentially harmful medications for older persons and are therefore recommended to be avoided among them [1]. Many psychotropics have anticholinergic properties [1]. Psychotropics are associated with an increased risk for serious adverse effects, such as falls, cerebrovascular events, and increased mortality [2–4]. In long-term care settings where dementia is prevalent, psychotropics often are prescribed off-label to manage neuropsychiatric symptoms (NPSs) related to dementia. However, first-line therapy is recommended to be non-pharmacological [5]. Despite the guidelines and limited evidence on effectiveness in the treatment of NPSs with psychotropics among older persons, psychotropic use and even psychotropic polypharmacy remain highly prevalent in long-term care settings [6–8].

Psychotropic medications can not only be harmful to older persons but can also incur financial costs. Potentially harmful medication use is associated with increased adverse effects, health care utilization, and costs [9–11]. In Finland, the overall consumption of psychotropics in 2019 was 150.8 defined daily doses (DDD)/1000 inhabitants/day, and an increasing trend has been observed during recent years [12]. The cost of psychotropics was approximately about 103 million Euros in 2019 [12]. The matter is also essential from the perspective of society, as the reimbursement rate of psychotropics is mainly 40% and individuals bear the remaining costs [13].

Several initiatives aim to reduce the use of psychotropics [14]. The approaches range from regularly conducted medication reviews by physicians or multidisciplinary teams for shared decision-making, multidisciplinary educational interventions, and professional support [14]. Several studies in nursing-home settings have shown that educational interventions are successful in reducing psychotropic drug use [15–18]. Furthermore, such interventions have led to fewer adverse effects [15]. However, despite the reduction in the number of psychotropics, there is no evidence of what kind of overall changes in the actual drug doses or costs have occurred.

To the best of our knowledge, most studies in long-term care have measured the temporal changes in psychotropic use as the number of drugs instead of actual dosages. Counting the relative proportions of defined daily doses (rDDDs) allows examination of the changes in the individual drug doses over time, even if the number of drugs remains stable. Thus, the total burden of psychotropics and sedation may decrease along with a decline in doses, even though the number of drugs used remains the same. Measuring the rDDD enables the comparison of various drug groups and analysis of drug costs.

The primary aims of the study were to investigate the use of potentially harmful medications (PHMs) in long-term care and to study whether staff training had an effect on the use of PHMs and related adverse effects. The results, which have been published previously, showed that training nurses in long-term care settings reduced the number of psychotropics and PHMs [15]. In the current study, we refined our analyses to explore the rDDDs. The aims of this study were to investigate the temporal changes in psychotropic use by rDDDs in the intervention and control group and to analyze the possible change in drug costs according to the changes in drug use as measured by rDDDs.

## 2. Methods

### 2.1. Study design

This study was a cluster-randomized controlled intervention trial in assisted living facilities in Helsinki during the years 2011 and 2012. The protocol is registered in the Australian New Zealand Clinical Trials Registry (ACTRN). Cluster randomization was used to avoid contamination of the intervention. All 36 assisted living facility units were assessed by using the Minimum Data Set (MDS)/Resident Assessment Instrument (RAI) version 2.0 for home care [19] and participating wards were selected. Wards were paired into 10 dyads with a case-mix as similar as possible. Of 320 residents in total, 93 refused or did not fulfill the inclusion criteria; 227 residents were included in the study. The units were randomized with computer-generated random numbers into intervention (118 subjects) or control (109 subjects) arms. The study was approved by the Ethics Committee of the Helsinki University Central Hospital.

### 2.2. Participants

Residents and their closest proxy received written information about the study and its course. Residents provided written informed consent to participate before starting any study procedures. The resident’s closest proxy provided written consent if the participant had significant cognitive decline (Mini-Mental State Examination [MMSE] < 20). The inclusion criteria were age ≥65 years and living permanently in an assisted living facility, native Finnish speaking, using at least one medication of any kind, and having estimated survival ≥6 months.

### 2.3. Measurements

Study nurses were registered nurses who received thorough training for the structured assessments. They were outside the wards and not aware of the outcome measures. They assessed the residents at baseline, 6 months, and 12 months. Study nurses were unaware of the intervention and unaware of all randomization procedures. Participants’ demographic data (including age, gender, and education) and diagnoses were retrieved from medical records. Comorbidity for each resident was calculated using the Charlson comorbidity index (CCI) [20]. Cognition was assessed by using the MMSE [21] and nutritional status was measured by the Mini Nutritional Assessment (MNA) [22].

Regularly used medications were retrieved from residents’ list of medicines on the day of the assessment. Drugs were classified using the Anatomical Therapeutic Chemical (ATC) classification system recommended by the World Health Organization [23]. Psychotropics included antipsychotics (ATC-N05A), antidepressants (ATC-N06A), anxiolytics (ATC-N05B), and hypnotics (ATC-N05C).

DDD corresponds to a standard unit of an average daily dose for a certain drug for its main indication for adults [23]. It should be noted, that DDD does not necessarily correspond to the most common dose or even indication used for older persons, as lower doses are in many cases recommended to be prescribed for older people. The prescribed daily dose is defined as the average dose prescribed according to a representative sample of prescriptions and can be determined from medical or pharmacy records [23]. The rDDD of each psychotropic was calculated by dividing the participants’ prescribed daily doses by the DDDs of the corresponding medication. This enables the comparison of various drug groups.

### 2.4. Intervention

The intervention was educational training provided by a geriatrician. The learning process was problem-based and learner-centered. During the first of two afternoons, the nursing staff of the intervention arms received education about changes in drug metabolism in old age, polypharmacy, renal failure in old age and use of the decision support database [24], common drug-drug interactions and use of the interaction database [25], and potentially harmful drugs in older persons. They also had group discussions about beneficial drugs in older persons, such as vitamin D [26] and anticoagulants in case of atrial fibrillation [27] in order to elaborate the education to focus not only on drug-related harms, but also to enlighten the importance of certain medication treatments, and further to achieve a comprehensive understanding of drug therapy of older persons. The second session was a case-based learning workshop. Each ward introduced two or three different drug-related problems their patients had experienced. These problems were discussed and solved together. The discussions in educational sessions were important reflections with respect to their own daily work and in line with the principles of adult education [15]. The staff also received a list of potentially harmful drugs. Nurses were encouraged to discuss their patients’ medication problems such as antipsychotics having adverse effects such as stroke, or any psychotropics having risk for falls with their consulting physicians. Two of three consulting physicians (GPs or geriatricians) working in each NH ward took part in the intervention as participants. The staff of the control wards received the same education after the study was completed.

### 2.5. Outcome measures

The primary outcome measure of this study was the change in the rDDDs of psychotropics (antipsychotics [ATC-N05A], antidepressants [ATC-N06A], anxiolytics [ATC-N05B], and hypnotics [ATC-N05C]) at the 12-month follow up.

The secondary outcome was the change in the annual cost of all psychotropics in rDDDs during the 12-month follow-up. Costs were calculated using the wholesale prices of DDDs [28] and multiplying that with rDDDs. The cost measure was the retail price of the medicine, which was based on the wholesale price list of drugs. The nationwide wholesale price of the medicine in question was multiplied by 1.6 to achieve an estimate of the retail price [28]. The prices were converted to the 2019 level using the consumer health index for health care. All costs are expressed in Euros in 2019 prices [12].

### 2.6. Statistical analyses

The data are presented as means with standard deviations (SD) or numbers with percentages. Statistical comparisons between groups were performed using *t*-test, Mann-Whitney *U* test, chi-square test, or Fisher’s exact test when appropriate. Longitudinal measures were analyzed using generalized estimating equations (GEE) models (unstructured correlation structure) with appropriate distribution and link function. Models (GEE) included age, sex, and comorbidities as a covariate. The bootstrap (10 000 replications) method was used when the theoretical distribution of the test statistics was unknown or in the case of violation of the assumptions (e.g. non-normality). The normality of variables was evaluated graphically and by the Shapiro-Wilk *W* test. The Stata 16.1, StataCorp LP (College Station, TX, USA) statistical package was used for the analysis.

## 3. Results

Of 307 eligible residents, 227 participated. The intervention group included 118 residents and the control group included 109 residents. The data collection has been described previously [15]. The mean participant age was 83 years (range 65–102) and most were females (71%). There were no differences between groups regarding sex, age, or mean the number of drugs used regularly. At baseline, the residents in the intervention group had a significantly higher number of comorbidities measured by the CCI (3.2 vs. 2.5) than those in the control group. Of the participants, 69% in the intervention and 80% in the control group used at least one psychotropic regularly. The proportions of participants using more than two psychotropics were 34% and 35%, respectively. The mean number of psychotropics used was 1.13 (intervention group) and 1.34 (control group) (Table 1).

**Table 1.** Baseline characteristics of participants.

	Intervention group (*N* = 118)	Control group (*N* = 109)	*p*-value
Females, *n* (%)	77 (65.3)	84 (77.1)	.050
Mean age, years (SD)	82.9 (7.5)	83.5 (6.9)	.41
MNA, *n* (%)			
<17, malnourished	19 (16.1)	25 (22.9)	.31
17–23.5, at risk for malnutrition	74 (62.7)	67 (61.5)	
>23.5, well-nourished	25 (21.2)	17 (15.6)	
CCI, mean (SD)	3.2 (2.0)	2.5 (1.8)	.004
MMSE, mean (SD)	8.8 (8.2)	10.0 (8.2)	.25
Number of drugs used regularly, mean (SD)	7.5 (2.8)	7.8 (3.1)	.79
Proportion using harmful medications*, %	83.1	71.6	.038
Mean number of harmful medications* (SD)	2.9 (1.8)	2.5 (1.7)	.28
Mean number of psychotropics (SD)	1.13 (.99)	1.34 (.99)	.11
Proportion using ≥1 psychotropic medications, %	69.0	79.8	.075
Proportion using >2 psychotropic medications, %	33.9	34.9	.30
Psychotropic rDDD, mean (SE)	0.71 (0.07)	0.80 (0.07)	.34
Proportion using ≥1 antipsychotics, %	40.7	46.8	.35
Antipsychotic rDDD, mean (SE)	0.17 (0.04)	0.19 (0.04)	.81
Proportion using ≥1 antidepressants, %	47.5	52.3	.47
Antidepressant rDDD, mean (SE)	0.45 (0.05)	0.43 (0.05)	.82
Proportion using ≥1 anxiolytics, %	9.3	15.6	.15
Anxiolytic rDDD, mean (SE)	0.03 (0.01)	0.06 (0.01)	.17
Proportion using ≥1 hypnotics, %	5.1	11.0	.10
Hypnotic rDDD, mean (SE)	0.05 (0.02)	0.12 (0.03)	.10
Mean annual cost of psychotropics (€) ** (95 % CI)	136 (108–166)	179 (139–230)	.13

MNA, Mini-Nutritional Assessment [22]; CCI, Charlson Comorbidity Index [20]; MMSE, Mini-Mental State Examination [21]; rDDD, relative proportion of defined daily dose; SD, standard deviation; SE, standard error; CI, confidence interval.

*Harmful medications were any of the following: Beers Criteria medications, anticholinergic medications, use of multiple psychotropic medications, NSAIDs, and PPIs. See Pitkälä et al. [15].

**Annual costs, retail price in 2019 euros.

At baseline, the mean rDDD of psychotropics was 0.71(intervention group) and 0.80 (control group). Antidepressants and antipsychotics were the two most prevalent psychotropic subgroups used by the participants, as 48% of the intervention group and 52% of the control group used antidepressants. The respective figures for antipsychotics were 41% and 47%. Anxiolytics (intervention 9%, control 16%) and hypnotics (intervention 5%, control 11%) were used to a lesser extent. The mean rDDD of antidepressants was higher, whereas the rDDDs of other subgroups were lower. The mean annual cost of psychotropics at baseline was 136 Euros in the intervention group and 179 Euros in the control group (*p* = 0.13) (Table 1).

### 3.1. Effect of the intervention on the rDDDs of psychotropics

**Figure 1.**
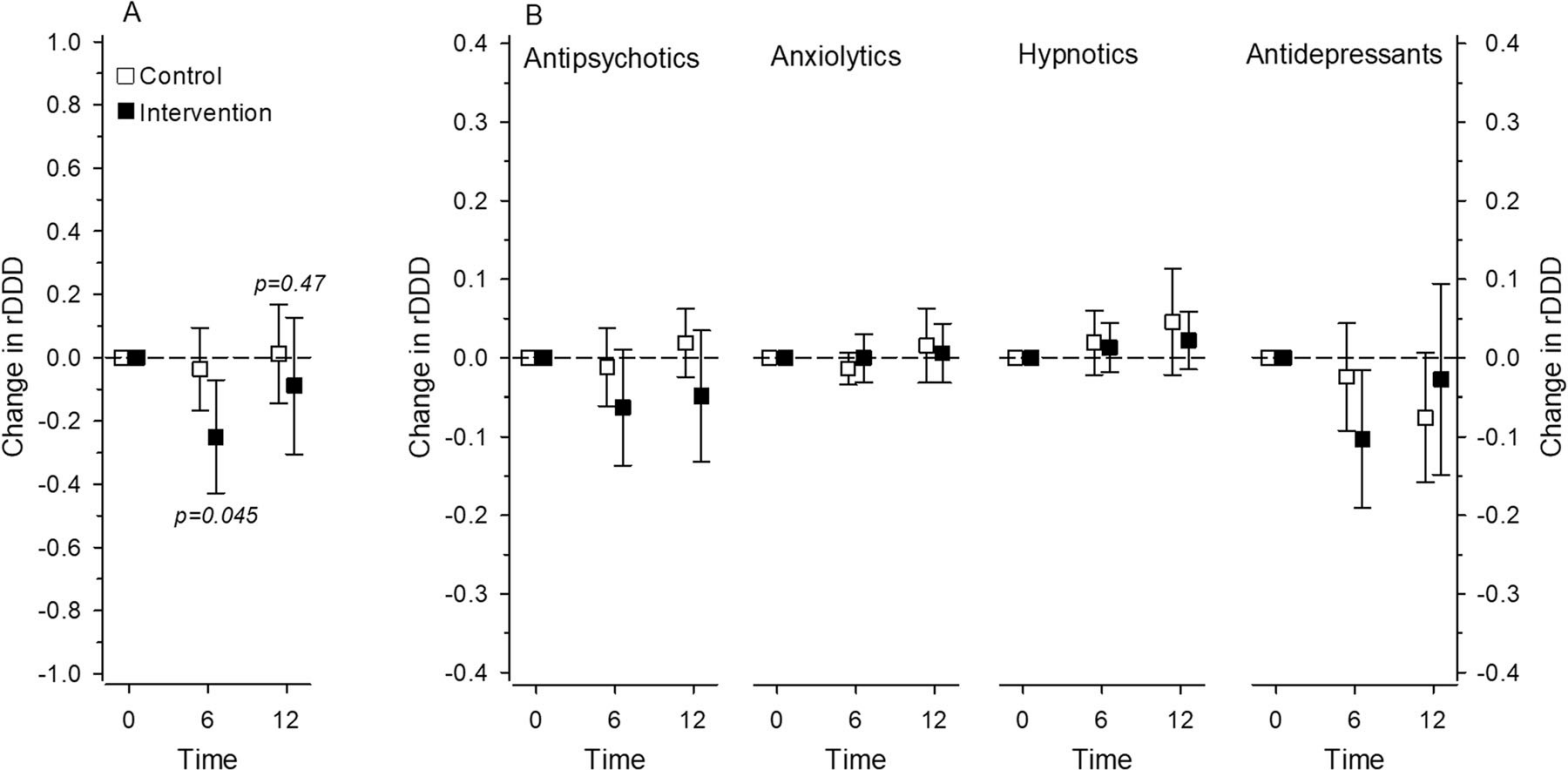
(A) Change in the relative proportions of DDDs (rDDDs) for all psychotropics. (B) Change in rDDDs in psychotropics analyzed by various subgroups. Adjusted for sex, age, and Charlson comorbidity index.

At 6 months follow up, a significant decrease in the rDDDs of all psychotropics was observed in the intervention group but not in the control group (*p* = 0.045; adjusted for age, sex, and CCI). However, the difference was no longer significant at 12 months (*p* = 0.47; adjusted for age, sex, and CCI). The rDDDs of psychotropics decreased slightly in the intervention group (−0.089; 95% CI −0.305 to 0.127) but remained more stable in the control group (0.012; 95% CI −0.143 to 0.167) (Figure 1A). When analyzing the subgroups of psychotropics, no significant differences were observed between the intervention and control groups (Figure 1B).

### 3.2. Effect of the intervention on the annual cost of psychotropics

**Figure 2.**
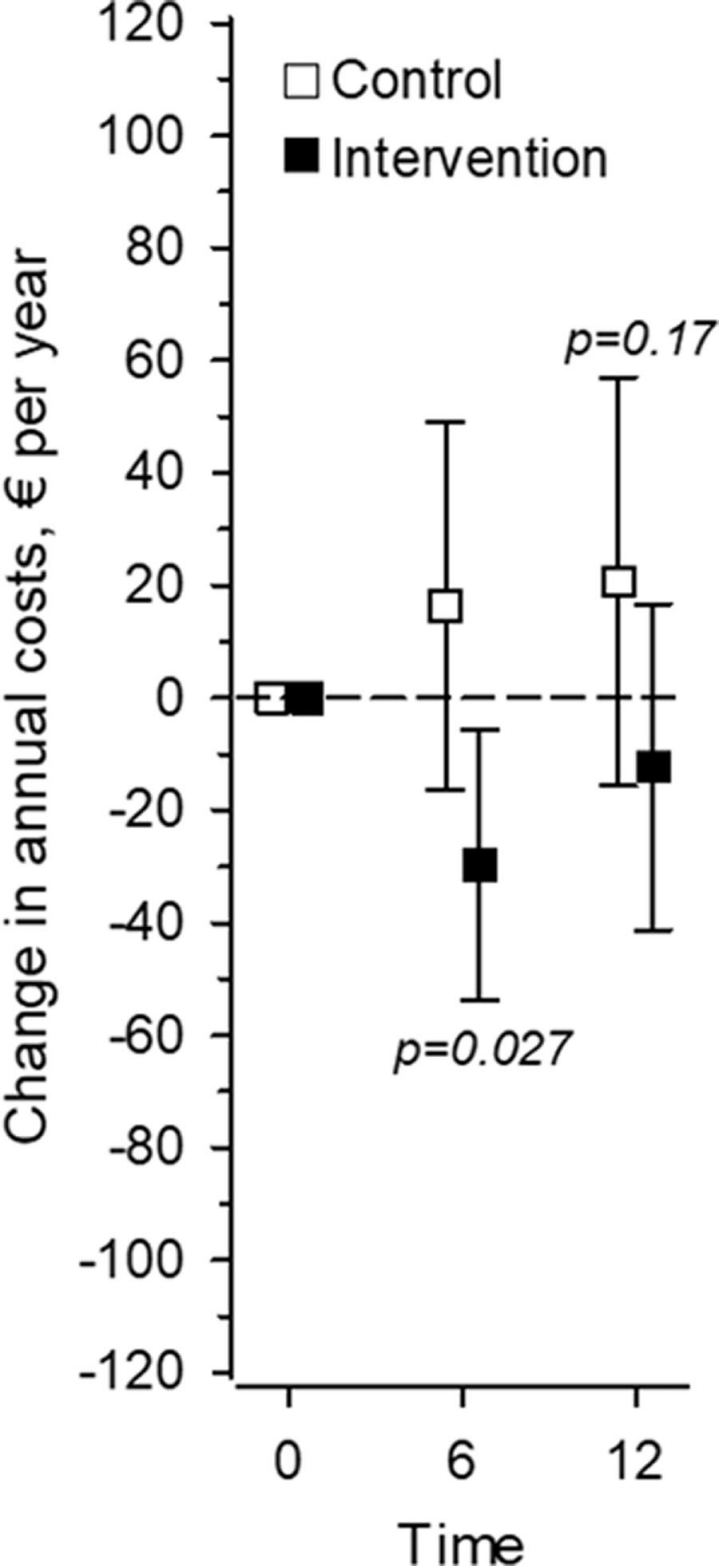
Change in annual cost (in Euros) of psychotropics. Adjusted for sex, age, and Charlson comorbidity index.

At 6 months of follow-up, the costs of psychotropic medications were significantly reduced in the intervention group but not in the control group (*p* = 0.027). At 12 months of follow-up, a significant difference was no longer observed between the groups (*p* = 0.17) (Figure 2). At 12 months, the annual cost of psychotropics decreased from a mean of 12.3 euros (95% CI −41.3 to 16.7) in the intervention group and increased from a mean of 20.6 euros (95% CI −15.8 to 57.0) in the control group. The cost savings for the intervention group (*n* = 118) were 1 450 euros in total. The decrease in the intervention group was 9% of the annual cost at baseline.

## 4. Discussion

According to our results, the educational intervention was successful in reducing the use of psychotropics (as assessed by rDDDs) in the short term. While this favorable effect could be observed at 6 months in the intervention group, no differences between the groups could be observed at 12 months. The same pattern was observed in the costs of psychotropics, as there was a significant reduction in costs at 6 months in the intervention group. To the best of our knowledge, this is the first study to assess the actual changes in the drug doses in this kind of intervention.

The rDDDs of antipsychotics, hypnotics, and anxiolytics were quite low even at baseline, indicating that these drugs were already prescribed at lower dosages than for younger adults. Hence, it is challenging to achieve differences through any intervention. However, the training intervention succeeded in decreasing the rDDDs, even though the baseline dosages were already low compared to middle-aged people. However, it must be emphasized that even low doses may be harmful to older people with respect to falls, cognitive decline and other adverse effects. *via* this intervention, which can be considered a feasible and minimally intense intervention, a reduction of over 20% in rDDDs of psychotropics was obtained at 6 months follow up. At 12 months the reduction had diminished and approached the level of the control group. The turnover of the nursing staff might partly explain this, as the education was not held continuously but only in sessions prior to the study. Despite the study protocol, it is possible that over time the educational information had also reached the facilities of the control group, causing a “dilution effect” that reduced the difference between the groups at 12 months.

Even though older and frail persons are prone to experience adverse effects of already lower drug doses compared with younger adults, thus far there is a lack of intervention studies that are focused on the changes in actual drug doses. In a previous study from the Netherlands, a dose-response relationship between psychotropics and fall risk was observed. The increased fall risk was already observed at low DDDs of psychotropics, and further increased with increasing DDDs of these drugs and their combinations [29].

While reducing the doses of psychotropics is essential considering their potentially harmful side effects, the reduction in medication costs is also important for individuals and for society. In this study, the medication costs decreased approximately 9% in the intervention group at 12 months. In contrast, the costs even increased in the control group, which indicates a favorable effect on medication costs, even though the difference between groups was not significant. These results are consistent with previous literature, as the use of potentially harmful medications is associated with higher health care costs [9–11]. In the study of Harrison and colleagues in an Australian long-term care population, two subgroups of psychotropics (antipsychotics and benzodiazepines) among the potentially inappropriate medications were responsible for most of the costs [11].

The strengths of this study include the real-life setting and a representative sample of older persons living in long-term care. Furthermore, the randomized, controlled trial design and meticulous counting of the doses and costs support the validity of our findings.

As a possible limitation, the data originates from 2011. However, we have converted the costs to the 2019 level. Secondly, this is not a cost-effectiveness analysis, which would provide a wider perspective of the changes in total health care costs. In addition, we did not have cost information of the psychotropic medications at the individual level. Therefore, the cost calculation is based on statistical averages of the annual costs of ATCs, which are based on the overall consumption of psychotropics [28]. On the other hand, this makes the results more generalizable as there are price differences between medicine brands. Since the reduction in a mean number of drugs does not necessarily consider the possible changes in the individual drug doses, the results from this study add to previous results.

## 5. Conclusions

Exposure to psychotropics was common in our population, as most residents in our study used at least one psychotropic drug. Cognitive impairment is highly prevalent among long-term care residents, which makes them more susceptible to the adverse effects of potentially harmful medications, such as psychotropics. One goal of care should be to prescribe wisely and avoid the use of potentially harmful medications to reduce adverse effects. The educational intervention can be considered successful in having achieved at least favorable short-term changes in psychotropic rDDDs. In addition, it was possible to observe cost savings. However, further research is needed to investigate whether and how the positive effects of the training could be maintained also after 6 months. Future research should focus on means for implementing the good practice of reducing the potentially inappropriate psychotropic burden. One way could be regular staff training instead of this kind of an occasional educational session.

## Ethics approval

The study has been approved by the Ethics Committee of Helsinki University Central Hospital.
